# Characterization of Brain Inflammation, Apoptosis, Hypoxia, Blood-Brain Barrier Integrity and Metabolism in Venezuelan Equine Encephalitis Virus (VEEV TC-83) Exposed Mice by In Vivo Positron Emission Tomography Imaging

**DOI:** 10.3390/v11111052

**Published:** 2019-11-13

**Authors:** Thomas M. Bocan, Robert G. Stafford, Jennifer L. Brown, Justice Akuoku Frimpong, Falguni Basuli, Bradley S. Hollidge, Xiang Zhang, Natarajan Raju, Rolf E. Swenson, Darci R. Smith

**Affiliations:** 1 Translational Sciences Directorate, Countermeasure Development Division, U.S. Army Medical Research Institute of Infectious Diseases, 1425 Porter St., Ft. Detrick, MD 21702, USA; robert.g.stafford2.civ@mail.mil; 2 Cherokee Nation Assurance, 777 West Cherokee Street, Catoosa, OK 74015, USA; 3 Foundational Sciences Directorate, U.S. Army Medical Research Institute of Infectious Diseases, 1425 Porter St., Ft. Detrick, MD 21702, USA; jennifer.l.brown436.ctr@mail.mil; 4 General Dynamics Information Technology (GDIT), 3211 Jermantown Road, Fairfax, VA 22030, USA; 5 Foundational Sciences Directorate, Virology Division, U.S. Army Medical Research Institute of Infectious Diseases, 1425 Porter St., Ft. Detrick, MD 21702, USA; justice.akuokufrimpong.ctr@mail.mil (J.A.F.); bradley.s.hollidge.ctr@mail.mil (B.S.H.); 6 Imaging Probe Development Center, National Heart, Lung, and Blood Institute, National Institutes of Health, Bethesda, MD 20892, USA; bhattacharyyaf@nhlbi.nih.gov (F.B.); xiang.zhang2@nih.gov (X.Z.); natarajan.raju@nih.gov (N.R.); rolf.swenson@nih.gov (R.E.S.); 7 Immunodiagnostic Department, Naval Medical Research Center, 8400 Research Plaza, Fort Detrick, MD 21702, USA; darci.r.smith.civ@mail.mil

**Keywords:** alphaviruses, Venezuelan equine encephalitis, VEEV TC-83, C3H/HeN mice, PET imaging, neuroinflammation, apoptosis, hypoxia, BBB integrity, glucose metabolism, [^18^F]DPA-714, [^18^F]CP-18, [^18^F]FMISO, [^18^F]albumin, [^18^F]FDG

## Abstract

Traditional pathogenesis studies of alphaviruses involves monitoring survival, viremia, and pathogen dissemination via serial necropsies; however, molecular imaging shifts this paradigm and provides a dynamic assessment of pathogen infection. Positron emission tomography (PET) with PET tracers targeted to study neuroinflammation (*N*,*N*-diethyl-2-[4-phenyl]-5,7-dimethylpyrazolo[1,5-a]pyrimidine-3-acetamide, [^18^F]DPA-714), apoptosis (caspase-3 substrate, [^18^F]CP-18), hypoxia (fluormisonidazole, [^18^F]FMISO), blood–brain barrier (BBB) integrity ([^18^F]albumin), and metabolism (fluorodeoxyglucose, [^18^F]FDG) was performed on C3H/HeN mice infected intranasally with 7000 plaque-forming units (PFU) of Venezuelan equine encephalitis virus (VEEV) TC-83. The main findings are as follows: (1) whole-brain [^18^F]DPA-714 and [^18^F]CP-18 uptake increased three-fold demonstrating, neuroinflammation and apoptosis, respectively; (2) [^18^F]albumin uptake increased by 25% across the brain demonstrating an altered BBB; (3) [^18^F]FMISO uptake increased by 50% across the whole brain indicating hypoxic regions; (4) whole-brain [^18^F]FDG uptake was unaffected; (5) [^18^F]DPA-714 uptake in (a) cortex, thalamus, striatum, hypothalamus, and hippocampus increased through day seven and decreased by day 10 post exposure, (b) olfactory bulb increased at day three, peaked day seven, and decreased day 10, and (c) brain stem and cerebellum increased through day 10. In conclusion, intranasal exposure of C3H/HeN mice to VEEV TC-83 results in both time-dependent and regional increases in brain inflammation, apoptosis, and hypoxia, as well as modest decreases in BBB integrity; however, it has no effect on brain glucose metabolism.

## 1. Introduction

Venezuelan equine encephalitis virus (VEEV) is a positive-sense RNA virus (*Togaviridae*, *Alphavirus*) that is endemic to Central and South America. VEEV is maintained in nature by cycling between rodent hosts and mosquito vectors. Large-scale epizootics in horses and spillover epidemics in humans occurred in North, Central, and South America. VEEV is highly pathogenic for equines and humans, leading to significant mortality in equines and high morbidity in humans that can result in fatal encephalitis in ≤1% of cases [1]. From a biodefense perspective, VEEV poses a significant threat because of its high infectivity, ease of production, stability, potential for aerosolization, and consistent induction of debilitating disease [2]. The high number of reported cases of laboratory-acquired infections by VEEV highlights the susceptibility of humans to aerosol infection [3]. Despite the significant threat posed by VEEV, no Food and Drug Administration (FDA)-approved medical countermeasures exist to treat this potentially life-threatening infection.

Mice are highly susceptible to wild-type strains of VEEV, where replication and dissemination results in lymphotropic and neurotropic phases [4]. This mimics what is seen in both humans and horses, making mice a suitable model. However, mice are not an ideal model, due to their high susceptibility to fatal disease, with mortality often reaching 100%. Less susceptible VEEV mouse models were developed using attenuated strains such as VEEV TC-83, which is a vaccine strain derived from the highly pathogenic Trinidad Donkey strain. VEEV TC-83 and other attenuated VEEV strains can replicate in the brains of many mouse strains without causing mortality [5,6]. However, mortality is observed in C3H/HeN mice exposed by intranasal instillation, aerosol, or direct intracranial injection [5,7]. Several studies established the utility of the C3H/HeN mouse model to closely mimic virulent VEEV encephalitis and for the testing of therapeutic candidates [8,9]. VEEV TC-83 in the C3H/HeN mouse model was shown to induce encephalitis, cause 100% mortality by day 12, and infect the olfactory epithelium following aerosol administration. VEEV TC-83 was shown to spread through the brain, sparing the caudal regions of the brain. Wild-type VEEV infection of C3H/HeN mice results in similar encephalitis, causes 100% mortality in seven days, infects the olfactory neuroepithelium, and spreads to the caudal regions of the brain [10]. Infection of C3H/HeN mice with VEEV TC-83 via intranasal installation results in neuroinvasion and neurovirulence, thereby allowing examination of therapies for their ability to treat central nervous system (CNS) infection in a BSL-2 mouse model.

The paradigm of traditional animal model pathogenesis and drug evaluation studies in infectious diseases typically involves the monitoring of survival, viremia, and pathogen dissemination via serial necropsies. New technology in the form of molecular imaging tools shifts this paradigm and offers less invasive alternatives capable of providing a dynamic assessment of pathogen infection in real time. In vivo imaging approaches are widely used to characterize disease progression and drug efficacy in the fields of neuroscience, oncology, cardiology, and immunology, but the application of positron emission tomography (PET) imaging in infectious disease research is limited [11]. Optical imaging approaches are utilized to monitor the progression of viral infections using luciferase-based inserts into the virus; however, these studies are limited to assessing viral replication and not the pathophysiological consequences of infection. A major objective of the present study is to exploit molecular imaging technology with targeted PET tracers to specific biological functions to address key unanswered questions about the pathogenic mechanisms of VEEV TC-83-induced CNS disease.

Positron emission tomography with PET tracers targeted to study neuroinflammation, apoptosis, hypoxia, blood–brain barrier (BBB) integrity, and metabolism are available to better understand VEEV encephalitic disease development in animal models. PET tracers for the assessment of macrophage accumulation and microglia/astrocyte activation in the brain focus on monitoring the 18-kDa translocator protein (TSPO). The TSPO radiotracer, *N*,*N*-diethyl-2-[4-phenyl]-5,7-dimethylpyrazolo[1,5-a]pyrimidine-3-acetamide ([^18^F]DPA-714), was evaluated in animal models of epilepsy, stroke [12], quinolinic acid-induced striatal inflammation [13], inflammatory bowel disease [14], rheumatoid arthritis [15], and Zika virus (ZIKV) [16] infection to assess the degree of inflammation and specificity of binding of the tracer to inflammatory cells. For the detection of apoptosis, a novel PET tracer, [^18^F]CP-18, a caspase-3 substrate, was developed [17]. [^18^F]CP-18 was shown to bind to areas of apoptosis in the thymus of a mouse model administered dexamethasone, which is known to induce apoptosis [17]. The most often used PET tracer for detection of hypoxia within viable tissue is [^18^F]fluormisonidazole ([^18^F]FMISO) [18]. In viable cells that are hypoxic, the PET tracer is reduced to a reactive intermediate by cellular reductases, which covalently binds to thiol groups of intracellular proteins and accumulates in areas of hypoxia. With albumin being a major constituent of blood, radiolabeling of albumin with PET or single-photon emission computed tomography (SPECT) isotopes is a useful tool to evaluate vascular permeability. In normal animals, [^18^F]albumin is retained in the circulation and serves as a blood pool agent; however, with damage, extravascular [^18^F]albumin is noted and collocates with regions of inflammatory cell infiltrates [19]. The metabolic tracer, [^18^F]fluorodeoxyglucose ([^18^F]FDG), is routinely used to assess tissue metabolism. In cases of Alzheimer’s disease and mild cognitive impairment, reductions in glucose metabolism as assessed by PET were noted [20]. The hypometabolism is believed to be associated with reduced neuronal activity and neuronal loss [21,22]. In cases of alphavirus infection and altered activity of neurons, one could use [^18^F]FDG to demonstrate overall changes in brain metabolism. Thus, application of these target-specific tracers may better define early cellular changes that could precede overt functional changes, identify regions in the brain that are more severely affected, and serve as dynamic tools for assessment of drug efficacy and drug pharmacodynamics following VEEV TC-83 infection.

In the current study, we utilized PET to evaluate the effect of VEEV TC-83 intranasal exposure on brain inflammation, apoptosis, BBB integrity, hypoxia, and glucose metabolism at a fixed time point following exposure, i.e., seven days. To better understand the kinetics of neuroinflammation and brain metabolic activity, we further examined the dynamic changes in [^18^F]DPA-714 and [^18^F]FDG uptake over the course of 10 days. We hypothesized that, with the progression of encephalitis, there would be a temporal increase in [^18^F]DPA-714 uptake that would track with regions of infection and could represent activation of brain microglia and influx of peripheral inflammatory cells potentially associated with a compromised BBB integrity, i.e., [^18^F]albumin uptake. Associated with inflammation and brain damage, we speculated that measures of neuronal health, i.e., [^18^F]FDG uptake, would decrease while changes in apoptosis and hypoxia would increase. Overall, we believed PET imaging could provide a dynamic measure of the pathophysiological consequences of VEEV TC-83 infection and potentially delineate inter-related events in disease progression.

## 2. Materials and Methods

### 2.1. Experimental Design

Three separate experiments were performed on C3H/HeN mice to characterize the effect of VEEV TC-83 exposure on the brain. In study 1, PET tracers specific for the assessment of inflammation, apoptosis, hypoxia, and BBB integrity were evaluated in different groups of animals at a single timepoint of seven days post VEEV TC-83 exposure. In study 2, a dynamic assessment of neuroinflammation was performed at three, seven, and 10 days post VEEV TC-83 exposure. In this study, the same group of animals underwent PET imaging at each of the three timepoints. In study 3, brain glucose metabolism was assessed in a new set of animals at three, seven, and 10 days post VEEV TC-83 exposure and, as for the measures of neuroinflammation, the same animal underwent PET imaging at all three timepoints. In all studies, groups of 7–10 male, 20 g, 4–5-week-old C3H/HeN mice (Jackson Laboratories, Bar Harbor, ME USA) were intranasally administered either phosphate-buffered saline (PBS) vehicle or 7000 plaque-forming units (PFU) of polyethylene glycol (PEG)/NaCl-precipitated, sucrose-purified VEEV TC-83 in 40 µL, i.e., 20 µL/nostril. The intranasal exposure was performed by directly dropping 20 µL of PBS (1×) or VEEV TC-83 into each nostril. The dose of 7000 PFU VEEV TC-83 was based on the observations of others who compared the neurovirulence and tissue tropism in C3H/HeN and Balb/c mice [10]. The live-attenuated VEEV vaccine TC-83 (NDBR 102, Lot 4 Run 3) used in these studies was manufactured by the National Drug Company (Swiftwater, PA, USA) and passaged twice in BHK cells before sucrose purification. The mice were monitored 2–3 times daily for clinical signs of disease and survival. In study 1 at seven days post VEEV TC-83 exposure, the mice underwent PET imaging. The conscious mice under restraint were administered 250 µCi (9.25 MBq) of either [^18^F]DPA-714, [^18^F]CP-18, [^18^F]FMISO, or [^18^F]albumin via tail vein injection and then returned to their cages for a 1-h uptake phase. After the uptake phase, the mice were anesthetized by isoflurane and placed on the Siemens Inveon preclinical PET/SPECT/CT system (Siemens Medical Solutions, Knoxville, TN, USA). After the PET imaging session, the mice were euthanized and brains were removed for measurement of whole-brain VEEV TC-83 titers by plaque assay. In studies 2 and 3, the time course of inflammation and glucose metabolism was assessed. Vehicle- and VEEV TC-83-exposed mice (*n* = 8–10/group) were administered either 250 µCi (9.25 MBq) of [^18^F]DPA-714 or [^18^F]FDG (Cardinal Health Pharmacy, Baltimore, MD, USA) via tail vein injection and, after a 1-h uptake phase, they were anesthetized by isoflurane and placed on the Siemens Inveon preclinical PET/SPECT/CT system (Siemens Medical Solutions, Knoxville, TN, USA). The same cohorts of control- and VEEV TC-83-exposed mice underwent sequential PET imaging on day three, seven, and 10 post VEEV TC-83 exposure.

Research was conducted under a USAMRIID Institutional Animal Care and Use Committee (IACUC)-approved protocol AP16-051 approved on 11/5/2016 in compliance with the Animal Welfare Act, Public Health Service (PHS) Policy, and other Federal statutes and regulations relating to animals and experiments involving animals. The facility where the research was conducted is accredited by the Association for Assessment and Accreditation of Laboratory Animal Care, International and adheres to principles stated in the Guide for the Care and Use of Laboratory Animals, National Research Council, 2011.

### 2.2. Synthesis of [^18^F]DPA-714, [^18^F]CP-18, [^18^F]FMISO, and [^18^F]Albumin

[^18^F]DPA-714, [^18^F]CP-18, [^18^F]FMISO, and [^18^F]albumin were obtained from the Imaging Probe Development Center (NIH, Bethesda, MD, USA). [^18^F]DPA-714, [^18^F]FMISO, and [^18^F]albumin were synthesized as previously described [23,24,25,26].

[^18^F]CP-18 was radiolabeled as previously described [17] with several modifications. A mixture of K_2_CO_3_ (150 µL of 20 mg/mL) and K_222_ (10 mg in 1 mL of CH_3_CN) was added to the vial containing fluorine-18. The solution was evaporated to dryness under nitrogen and vacuum at 110 °C. The residue was further azeotropically dried with acetonitrile (3 × 500 µL). A solution of pent-4-yn-1-yl 4-methylbenzenesulfonate precursor (20 mg) in anhydrous acetonitrile (0.1 mL) was added to the azeotropically dried mixture of [^18^F]KF/K_222_ and heated at 110 °C for 5 min under microwave heating (fixed temperature). Volatiles were transferred at 120 °C using nitrogen to a pre-cooled (in dry ice-acetonitrile bath) vial containing 2–3 mg of peptide precursor in 0.3 mL of 0.1M CuSO_4_, 40 mg of sodium ascorbate, and 20 mg of Tris[(1-benzyl-1*H*-1,2,3-triazol-4-yl)methyl]amine (TBTA) in 1:1 dimethylformamide/methanol (DMF/MeOH) (0.2 mL). The reaction mixture was stirred at room temperature for 30 min and diluted with 4 mL of water. The product was purified by high-performance liquid chromatography (HPLC) using a semi-preparative column (condition: Phenomenex Gemini 5 µm C18 110 Å column (250 × 10 mm), flow rate: 4 mL/min, 15–25% A in B for 25 min: A = acetonitrile with 0.1% trifluoroacetic acid (TFA), B = water with 0.1% TFA, t*_R_* ≈ 15 min). The HPLC collected fraction was diluted with 30 mL of water and passed through a preconditioned (5 mL of ethanol, 10 mL of air, 10 mL of water) C-18 plus Sep-Pak (Waters). The Sep-pak was washed with 10 mL of water, and the product was eluted with 0.6 mL of ethanol, followed by 5.4 mL of PBS (1×). The quality of the product was checked by HPLC (condition: Phenomenex Gemini 5 µm C18 110 Å column 150 × 4.6 mm), flow rate 1 mL/min, eluent: 10–75% A in B for 10 min: A = acetonitrile with 0.1% TFA, B = water with 0.1% TFA, t*_R_* = 5.4 min). The radiochemical purity of the product was > 98% with a molar activity of 44–118 GBq/µmol (1200–3200 Ci/mmol, *n* = 6). The overall radiochemical yield (RCY) was 42–53% (decay corrected, *n* = 6) in 90 min. The identity of the product was confirmed by comparing its HPLC retention time with co-injected authentic non-radioactive standard.

**Figure 1:**
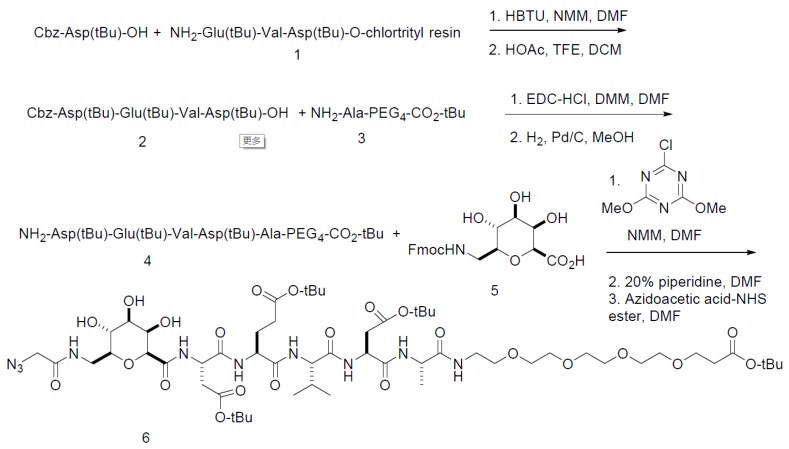
A Pictorial Representation of the Steps in the Synthesis of [^18^F]CP-18 (caspase-3 substrate).

The precursors for the synthesis of [^18^F]DPA-714 and the non-radioactive standards were synthesized as previously reported [23,24,26]. The precursor for [^18^F]CP-18 was synthesized with several changes from the published procedure [17] (Figure 1). The use of Cbz-Asp(*t*-Bu)-OH, the oxidation to prepare Fmoc-aminomethyl-galactose carboxylic acid, and coupling of the sugar to the peptide with chloro-3,5-dimethoxytriazine were novel. Briefly, Cbz-Asp(*t*-Bu)-OH was coupled to Glu(*t*-Bu)-V-D(*t*-Bu)-*O*-chlorotrityl resin (1) by solid-phase peptide synthesis using *N*-methyl morphine (NMM) and *N*,*N*,*N*’,*N*’-tramethyl-*O*-(1*H*-benzotriazol-1-yl) uranium hexafluorophosphate (HBTU) as the coupling agent in DMF. Mild acid cleavage of the tripeptide from the resin with acetic acid and trifluoroethanol (TFE) in dichloromethane (DCM) (2:2:6) liberated the C-terminal carboxylic acid, while maintaining the *t*-butyl side chain protecting groups. The tripeptide (2) was coupled to NH_2_-Ala-PEG_4_-CO_2_-tBu (3) with NMM and (3-dimethylaminopropyl)-*N*’ethylcarbodiimide hydrochloride (EDC-HCl) in DMF. Hydrogenolysis of the Cbz group with Pd/C liberated the N-terminus amine (4), which was coupled to Fmoc-aminomethyl galactose carboxylic acid (5) (prepared by the oxidation of Fmoc-aminomethyl-galactose with trichloroisocyanuric acid). Deprotection of the Fmoc group with piperidine followed by coupling to azidoacetic acid NHS ester in DMF completed the synthesis of the precursor (6). A non-radioactive ^19^F standard (CP18) was prepared as previously published [27]. The change from deprotecting Fmoc-DEVDA-PEG_4_-COO-*t*-Bu vs. Cbz-DEVDA-PEG_4_-COO-*t*-Bu via hydrogenation led to a simple filtration for isolation of the pure material, which resulted in a substantially higher yield. The second improvement was the coupling of 2-deoxy-2-Fmoc-aminomethylgalactonic acid to the peptide amine. The new procedure produced a purer product that could be isolated by column chromatography, which assisted the synthesis and purification of the subsequent product, leading to higher yields in both steps.

The precursors for synthesis of [^18^F]FMISO and the non-radioactive standard were obtained from ABX GmbH (Radeberg, Germany). PBS 1× buffer (Gibco) was obtained from Life Technologies (Carlsbad, CA, USA). Normal saline was obtained from Quality Biological (Gaithersburg, MD, USA). PD10 MiniTrapTM columns were obtained from GE Healthcare Bioscience (Pittsburgh, PA, USA). All other chemicals and solvents were received from Millipore Sigma (St. Louis, MO, USA) and used without further purification. Fluorine-18 was obtained from the National Institutes of Health cyclotron facility (Bethesda, MD, USA). Chromafix 30-PS-HCO_3_ anion-exchange cartridge was purchased from Macherey-Nagel (Düren, Germany). Phenomenex Gemini 5 µm C18 110 Å columns (250 × 10 mm and 150 × 4.6 mm) were purchased from Phenomenex (Torrance, CA, USA). All other columns and Sep-Pak cartridges used in this synthesis were obtained from Agilent Technologies (Santa Clara, CA, USA) and Waters (Milford, MA, USA), respectively. Sep-Pak C-18 Plus was conditioned with 5 mL of ethanol, 10 mL of air, and 10 mL of water. Semi-prep HPLC purification and analytical HPLC analyses for radiochemical work were performed on an Agilent 1200 Series instrument equipped with multi-wavelength detectors.

### 2.3. Plaque Assay

Vero cells (ATCC, USA) were plated at 4 × 10^5^ cells/well in a six-well plate and incubated for 24 h at 37 °C, 5% CO_2_. Serial dilutions of samples were made in 1× Minimal Essential Media (MEM) with 1% penicillin/streptomycin and 10% heat-inactivated fetal bovine serum (FBS). Uninfected-control and serially diluted samples were incubated with the Vero cells for 1 h at 37 °C, 5% CO_2_ for virus adsorption. A 1:1 mixture of 1% (*w*/*v*) agarose and 2× Basal Medium Eagle with Earle’s Salts (EBME) solution containing 2× EBME, 10% heat inactivated FBS (FBS-HI), 2% penicillin/streptomycin, 50 µg/mL gentamicin, and 2.5 µg/mL Fungizone (amphotericin B) was added. After addition, the 0.5% agarose/EBME overlay was incubated at room temperature for 15 min to allow the overlay to solidify. Plates were incubated at 37 °C, 5% CO_2_. After 24 h, a secondary 0.5% agarose/EBME overlay with 4% Neutral Red solution was added. The Vero cells were incubated overnight with the overlay at 37 °C, 5% CO_2_ and the plaques were subsequently counted.

### 2.4. PET/CT Imaging and Data Acquisition

PET/CT scanning was performed using an Inveon preclinical PET/SPECT/CT system (Siemens Medical Solutions, Knoxville, TN, USA) with a spatial resolution of ~1.5 mm full width at half maximum at the center of the field of view. Mice were placed in a restrainer (Braintree Scientific, Inc. Braintree, MA, USA) and administered 250 µCi (9.25 MBq) of [^18^F]DPA-714, [^18^F]CP-18, [^18^F]FMISO, [^18^F]albumin, or [^18^F]FDG in ~150 µL of volume via intravenous (IV) tail vein injection and returned to their cages for a 1-h uptake phase. After an hour, the mice were anesthetized with isoflurane (4% induction, 2% maintenance) delivered in oxygen. The mice were positioned in the center of the PET field of view, and PET imaging was initiated for a period of 20 min. Upon completion of the PET imaging session, a 5-min CT scan (80 kV, 500 µA, 98 µm, and 360° rotation in 220 steps) was initiated. During all imaging procedures, animal respiration rate and body temperature were monitored and maintained using an M2M-BioVet™ small-animal physiological monitoring system (M2M imaging, Cleveland, OH, USA). When scanning was completed, mice undergoing serial imaging were returned to their cages. At the end of the study, anesthetized mice were euthanized by CO_2_ asphyxiation, and the brains were removed for assessment of brain VEEV TC-83 titers.

### 2.5. Image Reconstruction and Data Analysis

All image reconstructions were performed using the Siemens’ Inveon Acquisition Workplace v2.1 software package (Siemens Medical Solutions, Knoxville, TN, USA). Hounsfield unit (HU)-calibrated CT data were reconstructed using a Feldkamp reconstruction algorithm with a Shepp–Logan reconstruction filter, slight noise reduction, and beam hardening correction applied. Decay-corrected PET images were reconstructed using an iterative Ordered Subset Expectation Maximization in 3 Dimensions/Maximum a Posteriori reconstruction (OSEM3D/MAP) algorithm with scatter, dead time, and CT-based attenuation correction. The reconstruction parameters included 18 subsets, with three iterations in a 128 × 128 matrix with a target resolution of 1.2 mm. PET images were co-registered to corresponding CT data using VivoQuant v2.5 image processing software (inviCRO, LLC, Boston, MA, USA) and were subsequently co-registered to a three-dimensional (3D) mouse brain atlas (included in VivoQuant software package) so that brain [^18^F]DPA-714, [^18^F]CP-18, [^18^F]FMISO, [^18^F]albumin, or [^18^F]FDG biodistribution could be quantified. PET imaging data were reported in terms of percentage injected dose per gram of tissue (%ID/g), calculated as the ratio of tissue radioactivity concentration (Bq/g) at time of scan to total injected activity (Bq) at time of scan.

### 2.6. Statistical Analysis

Statistical analysis was carried out using GraphPad Prism version 8.1.2. All values are means ± standard error of the mean (SEM), with a testing level (α) of 0.05 and adjusted *p*-values as indicated, where *p* < 0.05 signifies statistical significance. One-way ANOVA with post hoc paired two-tailed *t*-tests were used to compare each treatment group by day. One-way ANOVA with post hoc unpaired two-tailed *t*-tests were used to compare between treatment groups by day. A linear regression analysis was performed to assess the relationship between brain VEEV TC-83 titers and measures of [^18^F]DPA-714, [^18^F]CP-18, [^18^F]FMISO, and [^18^F]albumin uptake. 

## 3. Results

Following intranasal exposure to 7000 PFU VEEV TC-83, all animals survived until day three, with 10% mortality at day seven and 30% mortality at day 10. At day three, the mice appeared normal with no adverse behavior or grooming deficiencies. At day seven, the mice were subdued but reacted upon stimulation and appeared less groomed. By day 10, the mice appeared lethargic, less well-groomed, and subdued even after stimulation.

**Figure 2:**
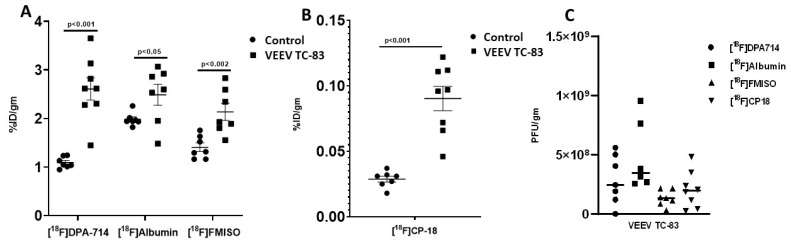
Positron emission tomography (PET) radiotracer uptake and Venezuelan equine encephalitis virus (VEEV) TC-83 titers in the brain. The measurements were made at seven days post VEEV TC-83 exposure (study 1; *n* = 7–10 animals/group). (**A**) [^18^F]DPA-714 (*N*,*N*-diethyl-2-[4-phenyl]-5,7-dimethylpyrazolo[1,5-a]pyrimidine-3-acetamide), [^18^F]FMISO (fluormisonidazole), and [^18^F]albumin uptake in the brain. (**B**) [^18^F]CP-18 uptake in the brain (Note: change in *y*-axis scale). (**C**) VEEV TC-83 titers in the brains of animals used in each of the PET studies.

**Figure 3:**
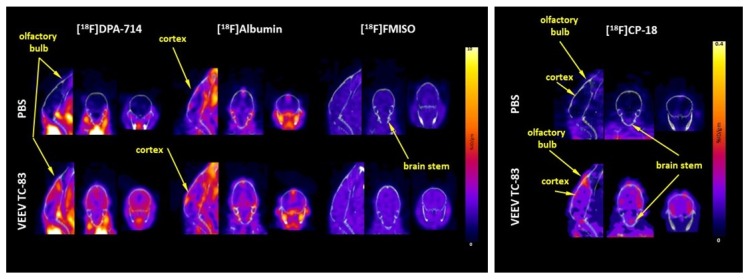
Representative PET images. PET images of the [^18^F]DPA-714, [^18^F]FMISO, [^18^F]albumin, and [^18^F]CP-18 uptake in the brain are depicted. Calibration bar represents percentage injected dose per gram of tissue (%ID/g) with blue = 0 and yellow/white = 10 (Note: the calibration bar for [^18^F]CP-18 ranges from 0 to 0.4 %ID/g rather than 0 to 10 %ID/g).

PET imaging at day seven with the four different PET tracers revealed marked increases in whole-brain inflammation and apoptosis and modest increases in albumin uptake and hypoxia. Mean [^18^F]DPA-714 uptake, a measure of inflammation, in the whole brain increased 2.4-fold with the greatest changes of 3–3.5-fold occurring in the olfactory bulbs, thalamus, and striatum (Figure 2 and Figure 3). 

Minor changes in [^18^F]DPA-714 uptake were noted in the brain stem and cerebellum, i.e., 1.6–1.9-fold. Mean [^18^F]CP-18 uptake, a marker for apoptosis, in the whole brain increased three-fold (Figure 2 and Figure 3; Table 1). The most marked increases in [^18^F]CP-18 uptake of 4–5-fold occurred in the olfactory bulbs, cortex, thalamus, and striatum. Minor [^18^F]CP-18 uptake was seen in the brain stem and cerebellum, i.e., 1.5-fold. Mean [^18^F]albumin uptake, a measure of BBB integrity, increased by 25% across the entire brain (Figure 2 and Figure 3; Table 1). [^18^F]Albumin uptake increased in the cortex, thalamus, and striatum by an average of 31%, while no statistically significant difference was noted in other brain regions. Mean [^18^F]FMISO uptake, a marker of hypoxia, increased by 50% across the whole brain and subregions (Figure 2 and Figure 3; Table 1). 

Whole-brain VEEV TC-83 titer at day seven was 6.93 × 10^8^ ± 1.26 × 10^8^ PFU/g (mean ± SEM) (Figure 2). There were no statistically significant correlations between [^18^F]DPA-714, [^18^F]CP-18, [^18^F]albumin, or [^18^F]FMISO uptake and whole-brain VEEV TC-83 titers, i.e., Pearson *r* = 0.14–0.32 with *p* < 0.5–0.7.

**Figure 4:**
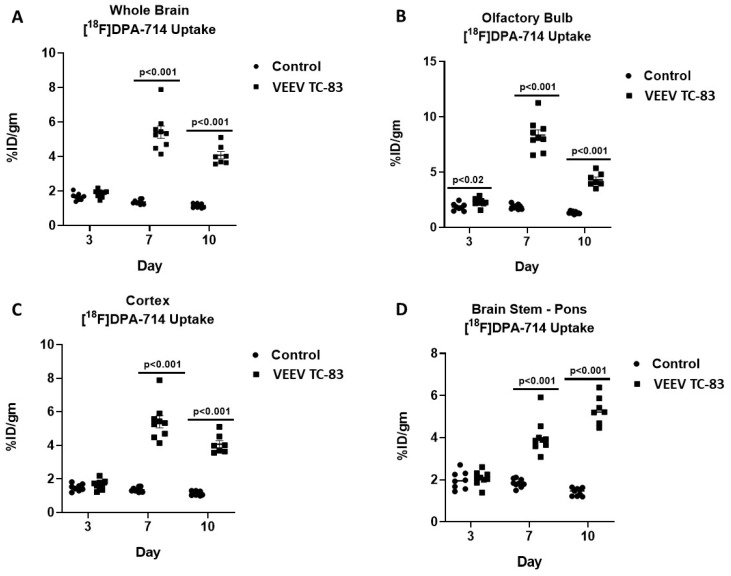
Time course of [^18^F]DPA-714 uptake. [^18^F]DPA-714 uptake was measured at three, seven, and 10 days post VEEV TC-83 exposure in whole brain, olfactory bulb, cerebral cortex, and brain stem (study 2; *n* = 8–10 animals/group). Lines represent statistically significant differences between respective day control and VEEV TC-83-exposed mice.

**Figure 5:**
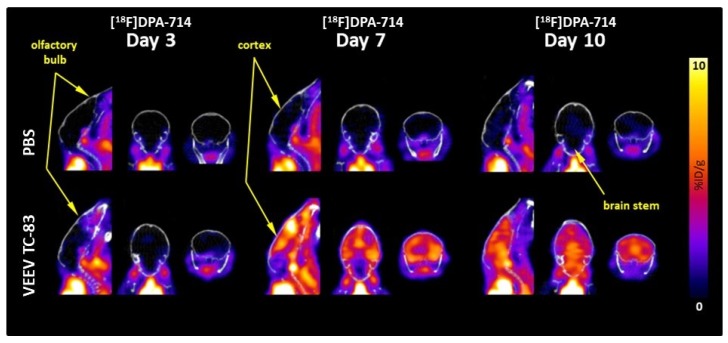
Representative PET images of [^18^F]DPA-714 uptake. The [^18^F]DPA-714 uptake in the whole brain of C3H/HeN mice at three, seven, and 10 days post intranasal exposure to 7000 plaque-forming units (PFU) of VEEV TC-83 is depicted. The calibration bar represents %ID/g with blue = 0 and yellow/white = 10.

Whole-brain regional and time-dependent changes in neuroinflammation were observed relative to uninfected controls over 10 days in mice exposed to VEEV TC-83. Over the course of the 10 days, the whole-brain [^18^F]DPA-714 uptake in uninfected controls was relatively constant at 1.48 ± 0.13 %ID/g (mean ± SEM) and there were no time- or brain region-dependent differences. Whole-brain [^18^F]DPA-714 uptake was unchanged at three days post VEEV TC-83 exposure but increased three-fold on both day seven and day 10 post exposure (Figure 4 and Figure 5). 

Olfactory bulb [^18^F]DPA-714 uptake significantly increased by 25% at day three, peaked at day seven, i.e., 4.5-fold elevation, and began decreasing at day 10, i.e., only three-fold elevation relative to controls (Figure 4 and Figure 5; Table 2). Cortical [^18^F]DPA-714 uptake was unchanged at day three but increased four-fold at day seven and 10 post exposure (Figure 4 and Figure 5; Table 2). Brain-stem [^18^F]DPA-714 uptake was unchanged at day three but increased two- and four-fold at day seven and 10, respectively (Figure 4 and Figure 5; Table 2). The magnitude and time course of changes in thalamus, striatum, hypothalamus, and hippocampus [^18^F]DPA-714 uptake were similar to those noted for the whole brain. Cerebellar [^18^F]DPA-714 uptake increased two-fold relative to controls.

Distinct time-dependent patterns of neuroinflammation were noted when comparing between time points for mice exposed to VEEV TC-83 (Figure 4 and Figure 5; Table 2). In comparison of day seven and 10 measures of [^18^F]DPA-714 uptake relative to day three, whole-brain [^18^F]DPA-714 uptake increased by 177% and 143%, respectively, but decreased by 11% between day seven and 10. Olfactory bulb [^18^F]DPA-714 uptake markedly increased by 270% at day seven relative to day three but declined by 47% between day seven and 10. Cortical [^18^F]DPA-714 uptake increased by 235% and 166% at day seven and 10 relative to day three, respectively, but, like the olfactory bulb, decreased by 20% between day seven and 10. In contrast, brain-stem [^18^F]DPA-714 uptake continued to increase by 102% and 176% at day seven and 10 relative to day three. Patterns of thalamus, striatum, hypothalamus, and hippocampus [^18^F]DPA-714 uptake were similar to whole brain. The pattern of cerebellar [^18^F]DPA-714 uptake was similar to brain stem although the magnitude was more modest, i.e., 54% and 97% at day seven and 10, respectively.

**Figure 6:**
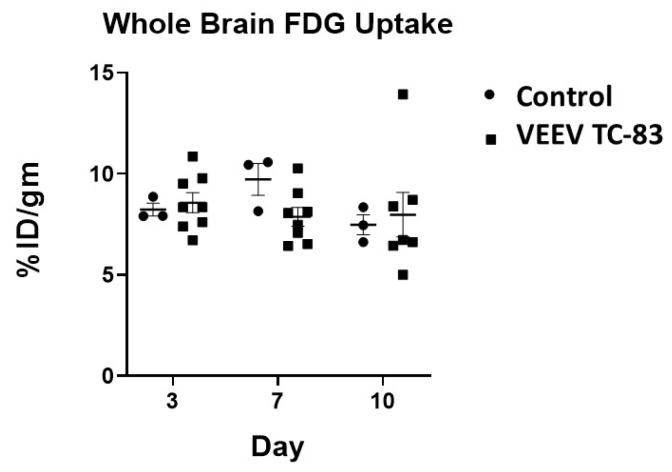
Time course of [^18^F]FDG (fluorodeoxyglucose) uptake. The [^18^F]FDG uptake at three, seven, and 10 days post VEEV TC-83 exposure in whole brain of C3H/HeN mice after intranasal exposure to 7000 PFU of VEEV TC-83 is shown (study 3; *n* = 8–10 animals/group). No statistically significant differences were noted relative to respective control or time point post VEEV TC-83 exposure.

**Figure 7:**
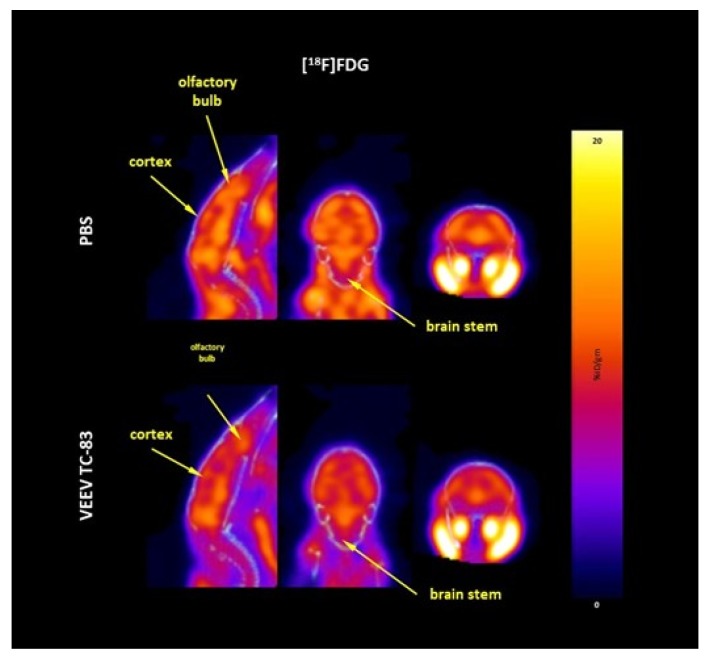
Representative PET images of [^18^F]FDG uptake. [^18^F]FDG uptake in the whole brain of C3H/HeN mice at seven days post intranasal exposure to 7000 PFU of VEEV TC-83. Similar images were seen at day three and 10 post VEEV TC-83 exposure. The calibration bar represents %ID/g with blue = 0 and yellow/white = 20 (Note: change in calibration bar scale).

Whole-brain glucose metabolism was unchanged following VEEV TC-83 exposure (Figure 6 and Figure 7). Whole-brain [^18^F]FDG uptake over the 10 days was 8.47 ± 0.44 and 8.14 ± 0.39 %ID/g (mean ± SEM) in control and VEEV TC-83-exposed mice, respectively. There were no time-dependent or regional differences in [^18^F]FDG uptake between control and VEEV TC-83-exposed mice (Table 3). 

## 4. Discussion

Intranasal exposure of C3H/HeN mice to 7000 PFU of VEEV TC-83 results in both time-dependent and regional increases in brain inflammation, apoptosis, and hypoxia, as well as modest decreases in BBB integrity, but has no effect on overall brain glucose metabolism. This conclusion is supported by the following findings of the current study: (1) whole-brain [^18^F]DPA-714 and [^18^F]CP-18 uptake increased three-fold; (2) [^18^F]albumin uptake increased by approximately 25% across the brain; (3) [^18^F]FMISO uptake increased by 50% across the whole brain and subregions; (4) whole-brain [^18^F]FDG uptake was unaffected by VEEV TC-83 exposure on day 3–10 post infection; (5) [^18^F]DPA-714 uptake in (a) cortex, thalamus, striatum, hypothalamus, and hippocampus increased through day seven and decreased by day 10 post exposure, (b) olfactory bulb increased at day three, peaked at day seven, and decreased by day 10, and (c) brain stem and cerebellum continued to rise through day 10. These data in totality demonstrate that PET imaging can dynamically follow disease progression in the absence of morbidity/mortality and that subtle brain sub-region changes can be detected, which might provide insight into the pathophysiologic mechanisms of alphavirus infection.

Inflammation is a hallmark of encephalitis and, as expected, VEEV TC-83 exposure induced a significant increase in [^18^F]DPA-714 uptake in the brain. We previously showed that the increase in [^18^F]DPA-714 uptake is correlated with increases in immunostaining for ionized calcium-binding adapter molecule 1 (IBA-1), a microglia/macrophage-specific calcium-binding protein which is highly upregulated during neuroinflammation, following ZIKV infection [16] in a mouse model. Given that [^18^F]DPA-714 binds to both brain-resident microglia and activated peripheral monocytes/macrophages, it is difficult to discern the involvement of each cell type in the overall uptake; however, assessment of the brain temporal differences might allow one to speculate as to the potential cellular involvement.

Microglial activation may precede an influx of peripheral inflammatory cells. Infection of the brain following intranasal exposure to alphaviruses was shown to occur through transit of the virus along the olfactory nerve into the olfactory bulb [27]. We observed that [^18^F]DPA-714 uptake was first to increase in the olfactory bulb at three days post exposure, prior to other brain regions. It is very likely that this early signal is due to activation of microglia. [^18^F]DPA-714 uptake markedly increased in the olfactory bulb and cortex at day seven and then declined by day 10, which may represent an influx of peripheral inflammatory cells and a potential near resolution of the inflammation. The changes in [^18^F]DPA-714 uptake lags seen in the brain stem and cerebellum, as well as the forebrain and mid-brain regions, may represent a lag in viral infection and/or replication or that the inflammatory response is more related to the influx of peripheral inflammatory cells and dependent on a breakdown in the BBB. The above speculation is consistent with the findings of others using different methods. One day after intranasal VEEV viral replicon particle instillation in mice, microglial proliferation was detected suggesting the development of microgliosis. Moreover, Cain et al. [28] found an increase in inflammatory monocytes at day two post infection in the olfactory bulb. In the cortex, increases in inflammatory cells beginning at day four post infection with a peak at day six were noted, which parallels the increase in BBB permeability in these brain regions as measured by sodium fluorescein extravasation. Our findings are consistent with the hypothesis that BBB integrity is critical for the influx of peripheral inflammatory cells as [^18^F]albumin was increased significantly in the cortex, thalamus, and striatum at day seven. In these same regions, [^18^F]DPA-714 uptake was increased at day seven and 10 post VEEV TC-83 exposure, but not at day three. Taken together, these studies support the hypothesis that microglia mediate early neuroinflammation with peripheral inflammatory cells contributing later when they infiltrate a compromised BBB. To further assess the role of PET in assessing BBB integrity and the relationship to monocyte/macrophage influx, one could perform a dynamic time course using [^18^F]albumin. 

Associated with the marked increase in inflammation at day seven post VEEV TC-83 exposure was a significant increase in [^18^F]CP-18 uptake. VEEV was shown to induce apoptosis in vitro [29] and in vivo by histologic analysis [30]. Neurons are the primary target cells of encephalitic alphaviruses, where viral cytopathology plays a major role in CNS dysfunction. Additionally, increasing evidence suggests that fatal alphavirus encephalitis is mediated by the immune response to virus infection rather than direct virus replication [31]; however, this does not rule out the possibility that both inflammation and direct viral mediated neuronal damage occur. There may also be a relationship between the degree of inflammation noted at day seven and apoptosis because inflammatory cytokines were noted to increase cytotoxicity [30]. Both [^18^F]DPA-714 and [^18^F]CP-18 demonstrate that involvement of microglia/macrophage activation and neuronal death are important in the progression of alphavirus pathology and that therapeutic interventions may differ depending upon the stage of disease progression. Based on these data, it is tempting to speculate that a direct anti-viral administered early to reduce the spread of virus across the brain and an anti-inflammatory agent to limit the involvement of peripheral mononuclear cells would reduce the brain damage and eventual clinical signs.

The lack of correlation between [^18^F]DPA-714, [^18^F]CP-18, [^18^F]albumin, or [^18^F]FMISO uptake and whole-brain VEEV TC-83 titers is somewhat perplexing but explainable. Brain VEEV TC-83 titers of 10^8^ PFU/g noted at day seven post exposure in the current study are consistent with published studies where C3H/HeN or C57/Bl6 mice were administered significantly higher doses of VEEV TC-83 intranasally, i.e., 10^7^ PFU [28,32,33]. Since the processes of inflammation, apoptosis, albumin uptake, and hypoxia may have different kinetics of expression, it is likely that one needs to study these correlations more dynamically. It is possible that albumin uptake may be an early event preceding the influx of inflammatory cells and that apoptosis and hypoxia may be late events after significant damage occurs. Without knowing the sequence of events a priori, it would be difficult to select time points for brain extraction and measurements of viral burden. In addition, given that the current study is a dynamic measure of inflammation and glucose metabolism in the same cohort of animals, a separate study with serial necropsies would be needed to assess brain viral titers. 

More important than the global changes in brain [^18^F]DPA-714, [^18^F]CP-18, [^18^F]albumin, or [^18^F]FMISO uptake and brain VEEV TC-83 titers are the regional changes which are time-dependent and may represent regional viral replication and resolution due to the inflammatory response or transit of viral signals across the neural network of the brain. Cain and colleagues mapped the changes in regional VEEV TC-83 titers across the brain following exposure to intranasal VEEV TC-83 [28]. As noted for the time course of changes in [^18^F]DPA-714 uptake described in the current study, peak brain titers of 10^9^–10^10^ PFU/g occurred in the olfactory bulb at day two, followed by the cortex at days 4–6, and then were reduced to near undetectable at day eight. The brain stem and cerebellar VEEV TC-83 titers were lower, i.e., 10^4^–10^6^ PFU/g, and were observed to rise throughout the eight days of the study. The pattern of changes in regional VEEV TC-83 titers is very similar to the patterns noted in the current study and suggest that brain [^18^F]DPA-714, [^18^F]CP-18, [^18^F]albumin, or [^18^F]FMISO uptake may be correlated at least in part with viral replication and clearance. 

The absence of changes in whole-brain and regional glucose metabolism may indicate that [^18^F]FDG PET may not be sensitive enough to detect subtle changes, or the brain metabolic activity is maintained despite infection; alternatively, it may reflect competing processes within the brain. For instance, the increases in inflammation shown by [^18^F]DPA-714 uptake would also produce an increase in [^18^F]FDG uptake, since inflammatory cells were shown to be highly metabolically active. The striking increase in [^18^F]CP-18 uptake may indicate that neurons and microglia undergo apoptosis, which would be reflected as a reduction in [^18^F]FDG uptake. Thus, the differences between inflammation and cell loss may be balanced or too subtle for [^18^F]FDG PET to detect a change. In order to assess whether there are deficits in neuronal glucose metabolism, it might be interesting to assess [^18^F]FDG metabolism in VEEV TC-83-infected mice following an evoked response to either an auditory, olfactory, or visual stimulus administered during the conscious uptake phase. 

Finally, measures of hypoxia may be another indicator of neuronal damage. [^18^F]DPA-714 uptake represents a dynamic increase in inflammation and microglia activation, which may result in brain damage as noted by increased apoptosis, as well as [^18^F]FMISO and [^18^F]albumin uptake. Since [^18^F]FMISO is reduced to a reactive intermediate by cellular reductases, which covalently binds to thiol groups of intracellular proteins and accumulates in areas of hypoxic viable cells, the observed uptake in [^18^F]FMISO uptake and the lack of change in [^18^F]FDG metabolism suggest that the brain cells are still viable. One might suspect that viral infection and replication across the brain promotes neuronal damage, which is reflected as apoptosis, increased areas of hypoxia, and a leaky microvasculature, as well as potentially reduced oxygenation of the neurons and brain parenchyma. It is also possible that the increase in [^18^F]FMISO uptake and hypoxia may be a consequence of reduced cerebral perfusion; however, this is not likely given the lack of change in [^18^F]FDG metabolism, where one would suspect that reduced perfusion would also result in a reduced glucose delivery to the brain, which would be reflected as a decrease in metabolism. To more fully address the relationship between perfusion and hypoxia, one could perform a dynamic PET study utilizing [^18^F]albumin and monitoring the early changes in brain perfusion post tracer injection.

In conclusion, intranasal exposure of C3H/HeN mice to VEEV TC-83 results in both time-dependent and regional increases in brain inflammation, apoptosis, and hypoxia, as well as modest decreases in BBB integrity, but has no effect on overall brain glucose metabolism. The current investigation also demonstrates that PET imaging with specifically targeted PET tracers can assess the pathology of alphavirus infection dynamically, in the same cohort of animals, and it can provide potential insights into the pathophysiologic cellular and brain responses.

## Figures and Tables

**Table 1:** Brain regional differences in [^18^F]DPA-714 (*N*,*N*-diethyl-2-[4-phenyl]-5,7-dimethylpyrazolo[1,5-a]pyrimidine-3-acetamide), [^18^F]CP-18 (caspase-3 substrate), [^18^F]albumin, and [^18^F]FMISO (fluormisonidazole) uptake. VEEV—Venezuelan equine encephalitis virus.

	Olfactory Bulb	Cerebral Cortex	Thalamus	Hypothalamus	Hippocampus	Striatum	Pons	Medulla	Cerebellum
[^18^F]DPA-714 uptake									
Control	0.933 ± 0.056	1.005 ± 0.048	0.785 ± 0.049	1.452 ± 0.064	1.041 ± 0.038	0.832 ± 0.041	1.360 ± 0.096	1.349 ± 0.097	1.697 ± 0.026
VEEV TC-83	3.156 ± 0.349 *	2.696 ± 0.273 *	2.644 ± 0.274 *	2.875 ± 0.250 *	2.734 ± 0.231 *	2.575 ± 0.272 *	2.634 ± 0.265 *	2.212 ± 0.162 *	2.342 ± 0.108 *
[^18^F]CP-18 uptake									
Control	0.0299 ± 0.002	0.0248 ± 0.003	0.0168 ± 0.003	0.0335 ± 0.003	0.0298 ± 0.002	0.0217 ± 0.002	0.0368 ± 0.003	0.0513 ± 0.004	0.0366 ± 0.004
VEEV TC-83	0.1161 ± 0.011 *	0.1093 ± 0.008 *	0.0770 ± 0.011 *	0.0810 ± 0.015 *	0.0966 ± 0.010 *	0.0928 ± 0.008 *	0.0619 ± 0.012	0.0743 ± 0.013	0.0565 ± 0.011
[^18^F]Albumin uptake									
Control	2.838 ± 0.097	1.839 ± 0.047	1.549 ± 0.063	3.160 ± 0.136	2.190 ± 0.041	1.747 ± 0.050	2.244 ± 0.135	1.976 ± 0.079	1.923 ± 0.065
VEEV TC-83	3.537 ± 0.324	2.419 ± 0.220 *	2.090 ± 0.185 *	3.314 ± 0.227	2.618 ± 0.208	2.237 ± 0.208 *	2.867 ± 0.271	2.415 ± 0.232	2.241 ± 0.191
[^18^F]FMISO uptake									
Control	1.448 ± 0.082	1.386 ± 0.089	1.415 ± 0.087	1.508 ± 0.092	1.371 ± 0.091	1.446 ± 0.090	1.439 ± 0.093	1.433 ± 0.087	1.379 ± 0.083
VEEV TC-83	2.193 ± 0.182 *	2.164 ± 0.176 *	2.181 ± 0.180 *	2.209 ± 0.181 *	2.106 ± 0.169 *	2.214 ± 0.180 *	2.098 ± 0.174 *	2.053 ± 0.174 *	1.994 ± 0.169 *

The data for study 1 are expressed as means ± standard error of the mean (SEM) in percentage injected dose per gram of tissue (%ID/g); *n* = 7–10 animals/group. * Statistically significant difference at *p* < 0.05.

**Table 2:** Brain regional differences in [^18^F]DPA-714 uptake over day three, seven, and 10 post VEEV TC-83 exposure.

	Olfactory Bulb	Cerebral Cortex	Thalamus	Hypothalamus	Hippocampus	Striatum	Pons	Medulla	Cerebellum
Control									
Day 3	1.841 ± 0.116	1.488 ± 0.073	1.298 ± 0.074	2.534 ± 0.153	1.621 ± 0.059	1.456 ± 0.089	1.984 ± 0.150	2.081 ± 0.125	1.990 ± 0.047
Day 7	1.872 ± 0.072	1.352 ± 0.045	1.135 ± 0.049	2.409 ± 0.083	1.392 ± 0.057 ^†^	1.335 ± 0.046	1.842 ± 0.066	1.953 ± 0.068	1.814 ± 0.067
Day 10	1.325 ± 0.038 ^†,‡^	1.145 ± 0.041 ^†,‡^	1.102 ± 0.060 ^‡^	1.681 ± 0.060 ^†,‡^	1.237 ± 0.057 ^†,‡^	1.117 ± 0.036 ^†,‡^	1.423 ± 0.061 ^†,‡^	1.556 ± 0.067 ^†,‡^	1.580 ± 0.058 ^†,‡^
VEEV TC-83									
Day 3	2.286 ± 0.119 *	1.651 ± 0.095	1.509 ± 0.071	2.578 ± 0.116	1.889 ± 0.092 *	1.560 ± 0.070	2.072 ± 0.111	2.308 ± 0.101	2.216 ± 0.095
Day 7	8.347 ± 0.473 *^,†^	5.407 ± 0.361 *^,†^	4.735 ± 0.409 *^,†^	5.342 ± 0.354 *^,†^	5.685 ± 0.380 *^,†^	5.354 ± 0.327 *^,†^	4.052 ± 0.266 *^,†^	3.799 ± 0.200 *^,†^	3.395 ± 0.186 *^,†^
Day 10	4.303 ± 0.234 *^,†,‡^	4.070 ± 0.212 *^,†,‡^	4.598 ± 0.345 *^,†^	4.801 ± 0.279 *^,†^	4.288 ± 0.247 *^,†,‡^	3.912 ± 0.248 *^,†,‡^	5.321 ± 0.248 *^,†,‡^	4.970 ± 0.390 *^,†,‡^	4.057 ± 0.166 *^,†,‡^

The data for study 2 are expressed as means ± SEM in %ID/g; *n* = 8–10 animals/group. * statistically significant difference from respective day control at *p* < 0.05. ^†^ Statistically significant difference from day three of each respective treatment at *p* < 0.05, e.g., day seven VEEV TC-83 vs. day three VEEV TC-83. ^‡^ Statistically significant difference from day seven of each respective treatment at *p* < 0.05.

**Table 3:** Brain regional differences in [^18^F]FDG (fluorodeoxyglucose) uptake over day three, seven, and 10 post VEEV TC-83 exposure.

	Olfactory Bulb	Cerebral Cortex	Thalamus	Hypothalamus	Hippocampus	Striatum	Pons	Medulla	Cerebellum
Control									
Day 3	8.618 ± 0.257	8.416 ± 0.294	8.977 ± 0.360	7.011 ± 0.397	7.937 ± 0.366	8.035 ± 0.326	7.990 ± 0.364	7.780 ± 0.415	8.426 ± 0.398
Day 7	10.253 ± 0.809	9.741 ± 0.760	10.820 ± 0.863	8.315 ± 0.868	9.112 ± 0.749	9.979 ± 0.821	9.739 ± 0.871	9.380 ± 0.672	9.778 ± 0.852
Day 10	7.734 ± 0.297	7.298 ± 0.392	7.869 ± 0.471	6.883 ± 0.717	6.978 ± 0.448	7.197 ± 0.478	7.770 ± 0.655	7.456 ± 0.694	8.480 ± 0.743
VEEV TC-83									
Day 3	8.980 ± 0.493	8.595 ± 0.419	9.178 ± 0.538	7.388 ± 0.556	8.119 ± 0.478	8.345 ± 0.508	8.613 ± 0.620	8.479 ± 0.590	8.972 ± 0.553
Day 7	8.528 ± 0.404	7.815 ± 0.433	8.399 ± 0.540	6.963 ± 0.452	7.449 ± 0.491	7.789 ± 0.475	7.900 ± 0.512	7.741 ± 0.508	8.184 ± 0.500
Day 10	8.500 ± 1.081	8.123 ± 1.207	8.603 ± 1.344	6.718 ± 0.770	7.610 ± 1.084	7.866 ± 1.188	7.790 ± 1.051	7.381 ± 0.860	8.120 ± 0.866

The data for study 3 are expressed as means ± SEM in %ID/g; *n* = 8–10 animals/group. No statistically significant differences from respective day control at *p* < 0.05 were noted.

## References

[1] Zacks M.A., Paessler S. (2010). Encephalitic alphaviruses. Vet. Microbiol..

[2] Franz D.R., Jahrling P.B., McClain D.J., Hoover D.L., Byme W.R., Pavlin J.A., Christopher G.W., Cieslak T.J., Friedlander A.M., Eitzen E.M. (2001). Clinical recognition and management of patients exposed to biological warfare agents. Clin. Lab. Med..

[3] Sewell D.L. (1995). Laboratory-associated infections and biosafety. Clin. Microbiol. Rev..

[4] Gleiser C.A., Gochenour W.S., Berge T.O., Tigertt W.D. (1962). The comparative pathology of experimental Venezuelan equine encephalomyelitis infection in different animal hosts. J. Infect. Dis..

[5] Ludwig G.V., Turell M.J., Vogel P., Kondig J.P., Kell W.K., Smith J.F., Pratt W.D. (2001). Comparative neurovirulence of attenuated and non-attenuated strains of Venezuelan equine encephalitis virus in mice. Am. J. Trop. Med. Hyg..

[6] Schoneboom B.A., Catlin K.M., Marty A.M., Grieder F.B. (2000). Inflammation is a component of neurodegeneration in response to Venezuelan equine encephalitis virus infection in mice. J. Neuroimmunol..

[7] Hart M.K., Pratt W., Panelo F., Tammariello R., Dertzbaugh M. (1997). Venezuelan equine encephalitis virus vaccines induce mucosal IgA responses and protection from airborne infection in BALB/c, but not C3H/HeN mice. Vaccine.

[8] Julander J.G., Bowen R.A., Rao J.R., Day C., Shafer K., Smee D.F., Morrey J.D., Chu C.K. (2008). Treatment of Venezuelan equine encephalitis virus infection with (-)-carbodine. Antivir. Res..

[9] Julander J.G., Skirpstunas R., Siddharthan V., Shafer K., Hoopes J.D., Smee D.F., Morrey J.D. (2008). C3H/HeN mouse model for the evaluation of antiviral agents for the treatment of Venezuelan equine encephalitis virus infection. Antivir. Res..

[10] Steele K.E., Davis K.J., Stephan K., Kell W., Vogel P., Hart M.K. (1998). Comparative neurovirulence and tissue tropism of wild-type and attenuated strains of Venezuelan equine encephalitis virus administered by aerosol in C3H/HeN and BALB/c mice. Vet. Pathol..

[11] Bocan T.M., Panchal R.G., Bavari S. (2015). Applications of in vivo imaging in the evaluation of the pathophysiology of viral and bacterial infections and in development of countermeasures to BSL3/4 pathogens. Mol. Imaging Biol..

[12] Harhausen D., Sudmann V., Khojasteh U., Muller J., Zille M., Graham K., Thiele A., Dyrks T., Dimaql U., Wunder A. (2013). Specific imaging of inflammation with the 18 kDa translocator protein ligand DPA-714 in animal models of epilepsy and stroke. PLoS ONE.

[13] Lavisse S., Inoue K., Jan C., Peyronneau M.A., Petit F., Goutal S., Dauquet J., Guillermier M., Dolle F., Rbah-Vidal L. (2015). [18F]DPA-714 PET imaging of translocator protein TSPO (18 kDa) in the normal and excitotoxically-lesioned nonhuman primate brain. Eur. J. Nucl. Med. Mol. Imaging.

[14] Bernards N., Pottier F., Theze B., Dolle F., Boisgard R. (2015). In vivo evaluation of inflammatory bowel disease with the aid of µPET and the translocator protein 18 kDa radioligand [18F]DPA-714. Mol. Imaging Biol..

[15] Pottier G., Bernards N., Dolle F., Boisgard R. (2014). [18F]DPA-714 as a biomarker for positron emission tomography imaging of rheumatoid arthritis in an animal model. Arthritis Res. Ther..

[16] Kuszpit K., Hollidge B.S., Zeng X., Stafford R.G., Daye S., Zhang X., Basuli F., Golden J.W., Swenson R.E., Smith D.R. (2018). [18F]DPA-714 PET imaging reveals global neuroinflammation in Zika virus-infected mice. Mol. Imaging Biol..

[17] Su H., Chen G., Gangadharmath U., Gomez L.F., Liang Q., Mu F., Mocharia V.P., Szardenings A.K., Walsh J.C., Xia C.F. (2013). Evaluation of [18F]-CP18 as a PET imaging tracer for apoptosis. Mol. Imaging Biol..

[18] Mees G., Dierckx R., Vangestel C., Van de Wiele C. (2009). Molecular imaging of hypoxia with radiolabelled agents. Eur. J. Nucl. Med. Mol. Imaging.

[19] Niu G., Lang L., Kiesewetter D.O., Ma Y., Sun Z., Guo N., Guo J., Wu C., Chen X. (2014). In Vivo Labeling of Serum Albumin for PET. J. Nucl. Med..

[20] Mosconi L. (2005). Brain glucose metabolism in the early and specific diagnosis of Alzheimer’s disease. FDG-PET studies in MCI and AD. Eur. J. Nucl. Med. Mol. Imaging.

[21] Herholz K. (2003). PET studies in dementia. Ann. Nucl. Med..

[22] Mielke R., Kessler J., Szelies B., Herholz K., Wienhard K., Heiss W.D. (1998). Normal and pathological aging--findings of positron-emission-tomography. J. Neural Transm. (Vienna).

[23] James M.L., Fulton R.R., Henderson D.J., Eberl S., Meikle S.R., Thomson S., Allan R.D., Dolle F., Fulham M.J., Kassiou M. (2005). Synthesis and in vivo evaluation of a novel peripheral benzodiazepine receptor PET radioligand. Bioorg. Med. Chem..

[24] Doorduin J., Klein H.C., Dierckx R.A., James M., Kassiou M., de Vries E.F. (2009). [11C]-DPA-713 and [18F]-DPA-714 as new PET tracers for TSPO: A comparison with [11C]-(R)-PK11195 in a rat model of herpes encephalitis. Mol. Imaging Biol..

[25] Oh S.J., Chi D.Y., Mosdzianowski C., Kim J.Y., Gil H.S., Kang S.H., Ryu J.S., Moon D.H. (2005). Fully automated synthesis of [18F]fluoromisonidazole using a conventional [18F]FDG module. Nuc. Med. Biol..

[26] Basuli F., Zhang X., Woodroofe C.C., Jagoda E.M., Choyke P.L., Swenson R.E. (2017). Fast indirect fluorine-18 labeling of protein/peptide using the useful 6-fluoronicotinic acid-2,3,5,6-tetrafluorophenyl prosthetic group: A method comparable to direct fluorination. J. Labelled Comp. Radiopharm..

[27] Sharma A., Knollmann-Ritschel B. (2019). Current understanding of the molecular basis of Venezuelan equine encephalitis virus pathogenesis and vaccine development. Viruses.

[28] Cain M.D., Salimi H., Gong Y., Yang L., Hamilton S.L., Heffernan J.R., Hou J., Miller M.J., Klein R.S. (2017). Virus entry and replication in the brain precedes blood-brain barrier disruption during intranasal infection. J. Neuroimmunol..

[29] Baer A., Lundberg L., Swales D., Waybright N., Pinkham C., Dinman J.D., Jacobs J.L., Kehn-Hall K. (2016). Venezuelan equine encephalitis virus induces apoptosis through the unfolded protein response activation of EGR1. J. Virol..

[30] Jackson A.C., Rossiter J.P. (1997). Apoptotic cell death is an important cause of neuronal injury in experimental Venezuelan equine encephalitis virus infection in mice. Acta Neuropathol..

[31] Steele K.E., Twenhafel N.A. (2010). Review Paper: Pathology of animal models of alphavirus encephalitis. Vet. Pathol..

[32] Poussard A., Patterson M., Taylor K., Seregin A., Jeanon S., Smith J., Salazar M., Paessler S. (2012). In vivo imaging systems (IVIS) detection of a neuro-invasive encephalitic virus. J. Visualized Expt..

[33] Taylor K., Kolokoltsova O., Ronca S.E., Estes M., Paessler S. (2017). Live, attenuated Venezuelan equine encephalitis virus vaccine (TC83) causes persistent brain infection in mice with non-functional αβ T-cells. Frontiers Microbiol..

